# ETH2/407: Ethical Problems in Non-Direct Patient-Physician Interaction

**DOI:** 10.2196/jmir.1.suppl1.e42

**Published:** 1999-09-19

**Authors:** G Papagounos, B Spyropoulos

**Affiliations:** ^1^Deree CollegeAthensGreece

**Keywords:** Medical Ethics, Responsibility, Patient-Physician Relation

## Abstract

The discussion of the ethical issues involved in patient-physician relations are traditionally based on the assumption that the parties involved are in direct contact and that their interaction occurs in the same spatio-temporal context. Problems such as confidentiality, paternalism, consent, responsibility etc. are discussed in light of the interpersonal interaction of the two parties. Medicine in the Internet and in other forms of digital medical applications, however, allow for a mode of interaction where the type of the contact between the patient and the physician is altered. The authors argue that the new types of physician-patient interaction create new ethical dilemmas.

The authors examine the traditional ethical issues in medicine and place them in their historical and methodological context. The immediacy factor in patient-physician relations is responsible for moral dilemmas which are non-existent in a "digital" environment. On the other hand, such an environment raises new issues and it creates new dilemmas. The concept of responsibility assumes a different moral weight in cases where the addressee of an action is distant in space and time. Requesting or granting consent is a different process in the Web than it is in an examination room of a hospital. Paternalistic behaviour on the part of a physician may well be viewed as committing the fallacy of authority by the patient when seeking medical information in the Internet.

In light of the above discussion, the authors claim that the major ethical issue arising out of the use of the Internet for medical purposes lies in the responsibility of both the physicians involved in the decision making process as well as the patients. The information made available on the Web by health care professionals is differently perceived by the various individuals accessing it. Further, the patient who engages in self-diagnosis on the basis of such information assumes a different type of responsibility from that when consulting a medical encyclopedia due to the interactive nature of the medium which attempts to minimise the assumption of "a universal type of patient" that underlies the whole enterprise.

It is therefore necessary to redefine the role of the patient and the physician in the Internet environment of health care services in order to determine the respective moral responsibility of both parties.

